# A Plant-Based Cholesterol-Lowering Diet Score Correlates with Serum LDL-Cholesterol Levels

**DOI:** 10.3390/nu16040495

**Published:** 2024-02-08

**Authors:** Jerry Polesel, Matteo Di Maso, Giovanna Esposito, Sara Vitale, Elvira Palumbo, Giuseppe Porciello, Ilaria Calabrese, Anita Minopoli, Bruna Grilli, Ernesta Cavalcanti, Diego Serraino, Egidio Celentano, David J. A. Jenkins, Livia S. A. Augustin

**Affiliations:** 1 Unit of Cancer Epidemiology, Centro di Riferimento Oncologico di Aviano (CRO) IRCCS, 33081 Aviano, Italy; polesel@cro.it (J.P.); serrainod@cro.it (D.S.); 2 Department of Clinical Sciences and Community Health, Branch of Medical Statistics, Biometry and Epidemiology “G.A. Maccacaro”, Università degli Studi di Milano, 20133 Milan, Italy; matteo.dimaso@unimi.it (M.D.M.); giovanna.esposito@unimi.it (G.E.); 3 Epidemiology and Biostatistics Unit, Istituto Nazionale Tumori, IRCCS “Fondazione G. Pascale”, 80131 Naples, Italyelvira.palumbo@istitutotumori.na.it (E.P.); g.porciello@istitutotumori.na.it (G.P.); e.celentano@istitutotumori.na.it (E.C.); 4 Healthcare Direction, “A. Cardarelli” Hospital, 80131 Naples, Italy; ilariacalabrese@live.it; 5 Laboratory Medicine Unit, Istituto Nazionale Tumori, IRCCS “Fondazione G. Pascale”, 80131 Naples, Italy; a.minopoli@istitutotumori.na.it (A.M.); b.grilli@istitutotumori.na.it (B.G.); e.cavalcanti@istitutotumori.na.it (E.C.); 6 Departments of Nutritional Science and Medicine, Faculty of Medicine, University of Toronto, Toronto, ON M5S 1A8, Canada; david.jenkins@utoronto.ca; 7 Clinical Nutrition and Risk Factor Modification Centre, St. Michael’s Hospital, Toronto, ON M5C 2T2, Canada; 8 Division of Endocrinology and Metabolism, Department of Medicine, St. Michael’s Hospital, Toronto, ON M5C 2T2, Canada; 9 Li Ka Shing Knowledge Institute, St. Michael’s Hospital, Toronto, ON M5C 2T2, Canada

**Keywords:** breast cancer, cholesterol, dietary pattern, lipid profile, plant-based diet

## Abstract

Background: A cholesterol-lowering diet score was previously developed for epidemiological studies; its association with serum lipid profile was not confirmed yet. Methods: The score was developed as an adaptation of the dietary portfolio for cholesterol reduction, assigning one point for adherence to seven dietary indicators and ranging from 0 (null adherence) to 7 (highest adherence). The score was calculated for breast cancer patients enrolled in the DEDiCa study using a 7-day food record; serum lipid profile, including total and low-density lipoprotein cholesterol (LDL-C), was evaluated in serum at baseline. Results: Patients with the highest adherence to the cholesterol-lowering diet (i.e., score ≥ 4) reported lower LDL-C level than women with score 0–1 (median: 107 mg/dL and 122 mg/dL, respectively; *p* < 0.01). The proportion of women with LDL-C above the recommended limit of 116 mg/dL was 60.0% with score 0–1 and 42.6% with score ≥4. Although the score directly correlates with consumption of foods from vegetal sources, it was mildly associated with the healthful plant-based diet index (r-Spearman = 0.51) and the Mediterranean Diet Adherence Screener (r-Spearman = 0.30) Conclusions: These results provide experimental evidence that the cholesterol-lowering diet score is capable of detecting a specific plant-based dietary pattern that affects circulating cholesterol levels.

## 1. Introduction

Serum cholesterol, in particular that contained in low-density lipoproteins (LDL-C), has been consistently linked to increased all-cause mortality, mainly due to higher risk of death from cardiovascular and heart diseases [1]. Evidence has also linked serum cholesterol to the onset of chronic metabolic disorders such as type 2 diabetes [2]. The association between serum cholesterol and cancer is more controversial: a direct association with increasing levels has been reported for colorectal cancer [3], high-grade prostate cancer [4], and pancreatic cancer [5], while results for breast cancer have been inconsistent [6,7].

Although dietary cholesterol has also been associated with an increased risk of several cancers [8], it is challenging to link cancer risk to serum cholesterol levels given that the two are poorly correlated [9]. It is well-known that serum cholesterol level depends on the lipid balance of endogenous hepatic cholesterol synthesis and clearance, as well as bile acid resorption, and that it may be influenced by dietary habits [10]. Notably, circulating serum cholesterol level, in particular LDL-C, is enhanced by the intake of different sources of fats—especially, cholesterol and saturated fatty acids (SFAs)—and it is reduced by the consumption of cholesterol-lowering foods and nutrients [11,12]. In a randomized controlled trial on patients with hypercholesterolemia, a one-month diet high in viscous fibers, plant sterols, soy food, and almonds induced a reduction of almost 30% of the level of serum total cholesterol and LDL-C, a reduction equivalent to that seen in the study arm undergoing statin use [13]. Similarly, low-glycemic index (GI) diets have been shown to reduce total cholesterol and LDL-C after four weeks in randomized controlled trials in comparison to high-GI diets [14].

In nutritional studies, however, it would be preferable to evaluate a dietary pattern rather than focusing on single markers of dietary intake. To this purpose, a score evaluating the adherence to a plant-based cholesterol-lowering portfolio diet has been recently developed [15], but its use is limited by the inclusion of foods (e.g., peanut butter, okra, oats) which are not staple foods in many diets around the world. Therefore, we aimed to develop a cholesterol-lowering diet score for epidemiological studies using dietary indicators that are easily derived from any type of diet, including the Mediterranean diet [16]. To this purpose, we considered seven dietary indicators that have been proven to lower circulating LDL-C [11,12,14]. The aim was to rank individual diets according to their ability to reduce serum cholesterol, under the assumption that the higher the score, the lower the level of circulating LDL-C. This would provide support for the use of the cholesterol-lowering diet score in etiological studies, especially when direct estimation of serum cholesterol levels is not possible. Therefore, using data from the DEDiCa trial [17], we evaluated the association between the cholesterol-lowering diet score and serum lipid profile, namely total cholesterol, LDL-C, high-density lipoprotein cholesterol (HDL-C), and triglycerides.

## 2. Materials and Methods

Data were derived from a multicenter randomized controlled trial on the effect of an intervention with dietary modification that increased physical activity and vitamin D supplementation (DEDiCa study) on recurrence in women with a breast cancer diagnosis [17]. Briefly, participants were 506 women aged 30–74 years who underwent surgery in the 12 months before enrolment for non-metastatic breast cancer (stages I–III), and who were followed up in seven national cancer institutes or oncologic departments of hospitals located in Southern and Northern Italy. Women who were unable to adhere to the study protocol or comprehend the consent form or were not willing to sign it were not included in the trial, nor were women with sarcoidosis or other granulomatous diseases or with hypercalcemia (i.e., serum calcium > 11 mg/dL), severe renal insufficiency or kidney stones (nephrocalcinosis or nephrolithiasis), AIDS, or other cancers.

Eligible women were randomized to two treatment arms: (i) standard care, which included a traditional Mediterranean diet, avoidance of sedentary behavior, and vitamin D supplementation to avoid insufficiency; (ii) high-intensity treatment, which included a low-GI traditional Mediterranean diet, plus 30 min of daily brisk walking, plus vitamin D supplementation to reach 60 ng/mL. At enrolment, blood pressure and body measurements were taken and each patient provided a fasting blood sample. Blood lipids (i.e., total cholesterol, LDL-C, HDL-C, and triglycerides) were measured directly in serum samples using commercially available standardized kits (Roche Diagnostics, Indianapolis, IN, USA) on an automatic instrument (Cobas C6000 analyzer—Roche Diagnostics) using Roche reagents, according to the manufacturer’s instructions. Specifically, total cholesterol was measured using the CHOL2 Cholesterol Gen.2 Cobas^®^ assay; HDL-cholesterol was measured using the HDLC4 HDL-Cholesterol Gen.4 Cobas^®^ assay; LDL-cholesterol was measured using the LDLC3 LDL-Cholesterol Gen.3 Cobas^®^ assay; and triglycerides were measured using the TRIGL Triglycerides Cobas^®^ assay [17].

Prior to randomization, baseline dietary habits were assessed through a 7-day food diary. Patients were asked to record any food and drink consumption during the week prior to the study baseline visit. The diary was then coded into a standardized food consumption form by trained nutritionists using a software for dietary analysis (Winfood, Medimatica S.R.L., Colonnella, Italy). Total energy and nutrient intake—including dietary fiber, monounsaturated fatty acids (MUFAs) from animal and vegetal sources, and SFAs—were then computed using the Italian food composition database [18]; GI values were derived from the Italian and International GI tables [19,20]. Physical activity was measured over one week prior to the baseline visit through a step counter with a 7-day memory (OMRON Walking Style IV, Omron Healthcare Italy, Lacchiarella, Italy) [17].

Adherence to a cholesterol-lowering diet was assessed through a score, previously developed to investigate the association between cholesterol-lowering diet and prostate cancer risk [16]. The score was created as an adaptation of the Portfolio diet for cholesterol reduction in patients with hypercholesterolemia [11,13], with the additional inclusion of GI [14]. Briefly, the score considered seven a priori dietary indicators derived from the food records: (i) high intake of soluble dietary fiber as a proxy of high viscous fibers; (ii) high MUFA intake, expressed as a percentage of total energy; (iii) high legume intake; (iv) low SFA intake, expressed as a percentage of total energy; (v) high intake of oil from seeds or corn as a proxy of phytosterols [21]; (vi) low dietary cholesterol intake; (vii) low GI foods. One point was assigned for each of the above-listed dietary indicators when it was fulfilled (i.e., when the indicator was above the cut-off for the cholesterol-lowering ones or when the indicator was below the cut-off for the cholesterol-raising ones), zero elsewhere. The final score was calculated as the sum of all points, and it theoretically ranged from 0 (no adherence) to 7 (complete adherence). The cut-offs previously identified in men [16] could not be used in this female population due to gender-specific dietary patterns. Therefore, the cut-off was set to identify for each parameter the women in the more favorable quartile for all dietary indicators, except for dietary cholesterol, where the recommended limit of <200 mg/day was chosen according to guidelines [22].

Considering that this is a “proof of concept” study aiming at validating the cholesterol-lowering diet score through the evaluation of the correlation between the proposed score and serum cholesterol levels, we inserted restriction parameters to limit potential sources of bias. First, considering the regional differences in dietary habits across study centers [23], the analysis was restricted to patients enrolled in one region only (i.e., Campania), which represents 74% of the whole study population (*n* = 374). Indeed, the low number of patients enrolled in Sicily (*n* = 54) and Friuli Venezia Giulia (*n* = 40) did not allow a reliable evaluation of regional differences, while controlling for individual patients’ characteristics such as age, body mass index (BMI), and physical activity. Furthermore, to avoid bias due to lipid-lowering medications, 82 women who were treated for hypercholesterolemia (*n* = 78) or hypertriglyceridemia (*n* = 4) at enrolment were excluded from the present analysis, thus leaving 292 participants eligible for the present analysis (median age: 50 years; Q_1_–Q_3_: 45–56 years).

Serum lipid profiles are reported as median values with first and third quartile (Q_1_–Q_3_). Differences across levels of cholesterol-lowering diet score were evaluated through the Kruskal–Wallis test. The trend in median values was tested through the Jonckheere–Terpstra test [24]. Furthermore, the proportions of patients beyond the recommended levels were calculated using the following cut-offs clinically recognized by the European Society of Cardiology and European Atherosclerosis Society [22]: 200 mg/dL for total cholesterol; 50 mg/dL for HDL-C; 116 mg/dL for LDL-C; and 150 mg/dL for triglycerides. Differences in such proportions across cholesterol-lowering diet score levels were evaluated through a χ^2^ test. To account for potential confounding of variables significantly associated with lipid profiles in the univariate analysis (i.e., age, BMI, tobacco smoking, and physical activity), the independent association between median serum cholesterol levels and potential predictors was evaluated though a quantile regression model. Specifically, quantile regression is a non-parametric extension of linear regression. Classical linear regression method enables estimating models for conditional means, while quantile regression is a technique for estimating models for conditional medians, and, in general, for the full range of other quantiles (e.g., 10th, 25th, 75th, or 95th percentile) [25]. We fit median regression models to estimate conditional medians of serum cholesterol levels according to the set of potential predictors. Significance of *β* coefficients was evaluated through Student’s *t*-test.

Finally, to evaluate whether the cholesterol-lowering diet score identifies a specific dietary pattern, the score was compared to two dietary patterns. Firstly, the adherence to the Mediterranean diet was estimated through the Mediterranean Diet Adherence Screener (MEDAS) [26]. MEDAS inquiries about the consumption of 14 typical and non-typical Mediterranean foods [27], thus ranging from 0 (lowest adherence) to 14 (highest adherence). Further, the adherence to a plant-based dietary pattern was estimated though the healthful plant-based diet index (hPDI) [28]. This score is based on 18 plant-based food groups, scoring the consumption of each food group with points equivalent to the quintile to which it belongs (e.g., 1 point when in the 1st quintile, 3 points when in the 3rd quintile, etc.). The 18 food group scores were then summed to a total, ranging from 18 (lowest adherence) to 90 (highest adherence). The cholesterol-lowering diet score was correlated to MEDAS and hPDI through the Spearman correlation coefficient r. Statistical significance was considered when *p* < 0.05 (two-tailed). Statistical analyses were conducted with R 4.2 software.

## 3. Results

Overall, 292 women met the inclusion criteria for the present analysis; 15.8% of them were diagnosed with stage III breast cancer (Table 1). Before trial intervention, 30.5% of women were overweight (i.e., BMI 25 to <30 kg/m^2^) and 30.1% were obese (i.e., BMI ≥ 30 kg/m^2^); low physical activity, defined as walking for <5000 steps/day, was reported by 47.6% of study participants. Serum total cholesterol, LDL-C, and triglyceride levels were significantly higher in women aged 50 years and older, in smokers, in patients with obesity, and in those who walked <5000 steps/day. Conversely, HDL-C level was higher in women with BMI <25 kg/m^2^ and in those who were physically active. No association was found with drinking habits and cancer stage.

For each dietary indicator contributing to the cholesterol-lowering diet score, cut-off and point allocation are reported in Table 2. Serum total cholesterol, LDL-C, and triglycerides were generally lower when each dietary indicator was met, though not statistically significant. When the seven dietary indicators were summed up to the cholesterol-lowering diet score, 47 women (16.1%) reported cholesterol-lowering diet score of 4–6, whereas 80 (27.4%) were scarcely adherent with scores of 0–1. None of the participants was fully adherent to a cholesterol-lowering diet, reaching the highest score (i.e., 7).

**Figure 1:**
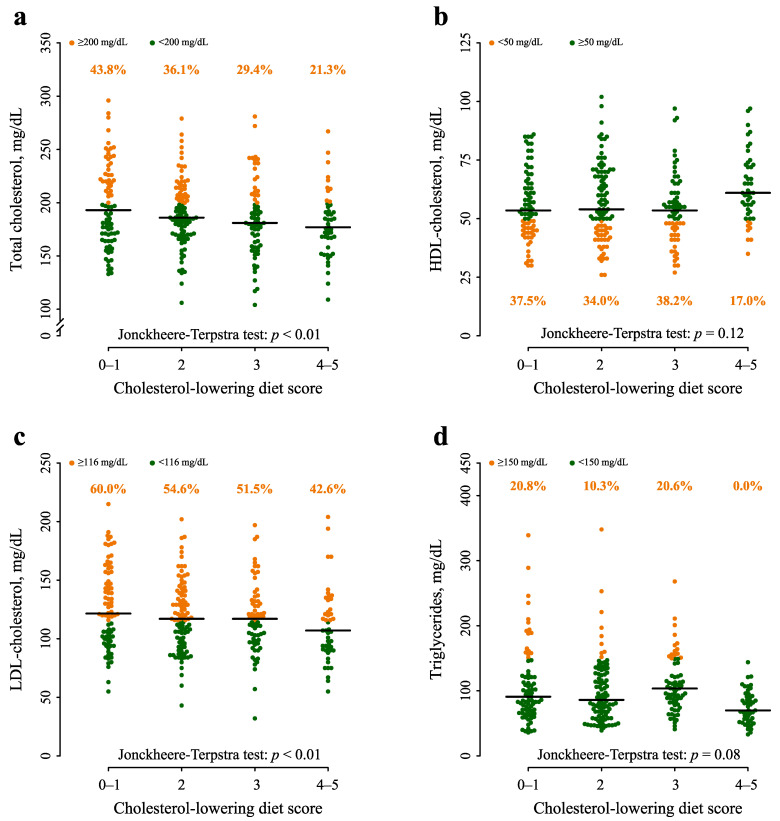
Serum levels of total cholesterol (**a**), high-density-lipoprotein (HDL) cholesterol (**b**), low-density-lipoprotein (LDL) cholesterol (**c**), and triglycerides (**d**) according to cholesterol-lowering diet score. Green and orange dots represent the values within and outside the recommended levels, respectively. Percentages in orange represent the proportion of patients above and below the recommended ranges. Horizontal bar represents the median value in each group.

Increasing cholesterol-lowering diet score was significantly correlated with lower levels of serum total cholesterol and LDL-C and with higher levels of HDL-C (Table 2 and Figure 1). Women with scores 4–6 reported the lowest total cholesterol (median: 177 mg/dL; Q_1_–Q_3_: 163–197 mg/dL; *p* < 0.01) and LDL-C levels (median: 107 mg/dL; Q_1_–Q_3_: 91–124 mg/dL), lower than women with score 0–1 (median total cholesterol: 193 mg/dL; Q_1_–Q_3_: 169–223 mg/dL, *p* < 0.01; median LDL-C: 122 mg/dL; Q_1_–Q_3_: 101–151 mg/dL, *p* < 0.01; Table 2). Notably, the proportion of women with serum total cholesterol above the recommended limit of 200 mg/dL declined from 43.8% in women with score 0–1 to 21.3% in women with score 4–6 (*p* < 0.01; Figure 1a). For LDL-C ≥116 mg/dL, the figures were 60.0% and 42.6%, respectively (*p* < 0.01; Figure 1c). No significant trend emerged for HDL-C and triglycerides; however, the proportion of women with the HDL-C < 50 md/dL was considerably lower for score 4–6 than for score 0–1 (i.e., 17.0% and 37.5%; χ^2^ test: *p* = 0.07). Notably, no patients with score 4–6 (0.0%) reported triglycerides ≥150 mg/dL compared to 20.8% of women with score 0–1 (χ^2^ test: *p* < 0.01).

To account for potential confounding due to socio-demographic characteristics and lifestyle factors, a median regression model was estimated for each component of the serum lipid profile (Table 3). Being overweight (i.e., BMI 25 to <30 kg/m^2^) or obese (i.e., BMI ≥ 30 kg/m^2^) was a significant and independent predictor of all components of the serum lipid profile. Accounting for differences due to age, smoking habits, BMI, and physical activity, the cholesterol-lowering diet score was confirmed to be inversely and independently associated with serum total and LDL-C level (*p* for trend: 0.02 and <0.01, respectively) and with triglyceride level (*p* for trend = 0.04).

The associations between the cholesterol-lowering diet score and intakes of food groups are investigated in Table 4. As expected, the daily intake of fruit, vegetables, whole grains, legumes, and olive oil increased with increasing adherence to the cholesterol-lowering diet (*p* < 0.01). Conversely, the cholesterol-lowering diet score was inversely associated to the consumption of refined grains, meat, and dairy products (*p* < 0.01). Despite the strong association with intake of food characterizing plant-based diets and the Mediterranean diet, the cholesterol-lowering diet score was mildly associated with the hPDI (r-Spearman = 0.51) and MEDAS (r-Spearman = 0.30). This indicates that the cholesterol-lowering diet score identifies a specific dietary pattern, different from the Mediterranean and the healthful plant-based patterns.

As a sensitivity analysis, the analyses were further conducted excluding women with other metabolic disorders, and the results were consistent (Table S1). Serum levels of total cholesterol and LDL-C significantly declined with increasing cholesterol-lowering diet scores even when women with diabetes mellitus and/or hyperglycemia were excluded from the analyses.

## 4. Discussion

The results of the present analyses support the association between the proposed cholesterol-lowering diet score and serum lipid profiles. Specifically, women with a score of ≥4 had the lowest serum levels of total cholesterol, LDL-C, and triglycerides. Notably, none of the food indicators significantly correlated with serum lipid profiles, supporting the role of a synergic dietary pattern rather than an effect due to a specific food. Therefore, the cholesterol-lowering diet score measures the adherence to a dietary pattern, different from other favorable patterns (e.g., the Mediterranean diet), which is capable of reducing cholesterol concentrations.

The dietary indicators in the score have been proven to reduce serum cholesterol levels individually [11,12,14,21] and combined within the Portfolio diet [12,13]. Water soluble fibers—such as β-glucans, pectins, and gums—reduce the reabsorption of cholesterol and bile acids in the small intestine by hindering the movement of bile acids into enteric micelles. This results in increased hepatic uptake of LDL particles needed to retrieve cholesterol and convert it into bile acids thus replenishing the liver bile acid pool [29]. Furthermore, dietary fiber from consumption of plant-based diets represents an indigestible carbohydrate and main source of energy for the gut microbiota which ferment dietary fiber to produce short chain fatty acids (SCFAs) such as propionate with cholesterol-lowering properties [30]. Phytosterols are a class of non-nutrients with cholesterol-lowering properties by virtue of reduced intestinal cholesterol absorption. They are found in oily plant foods such as seeds and in whole grains. In randomized controlled trials, a daily oral dose of 2 g of phytosterols, either sterols or stanols, has shown to reduce serum LDL-C by 9–13%, depending on age [31]. Phytosterols form insoluble crystals with cholesterol, limiting its incorporation into enteric micelles and inhibiting cholesterol transport proteins [29,31]. In addition, phytosterols have been demonstrated to competitively inhibit intestinal absorption of cholesterol [21,32], since both require the Nieman–Pick C1-Like 1 protein to enter the enterocytes [33]. Finally, diets characterized by low GI have been shown to decrease serum lipids, especially LDL-C, in randomized controlled trials [14]. Insulin is a stimulator of hydroxymethylglutaryl (HMG)-coenzyme A (CoA) reductase, a rate-limiting enzyme of hepatic cholesterol synthesis [34].

The main aim of this study was to test if the proposed cholesterol-lowering diet score was able to identify different levels of serum cholesterol and triglycerides. Although based on strong hypotheses, the score was derived from available data collected in observational studies, and the use of some proxies (e.g., soluble fibers for viscous fiber and seed/corn oil for phytosterol) could have hampered its validity. In addition, it is well known that retrospective studies are prone to selection and information bias, both increasing the chance of misclassification. Therefore, the use of dietary data collected in DEDiCa trial, including a 7-day food record prior to dietary intervention and blood samples for cholesterol analysis, was an opportunity to validate our score.

The present cholesterol-lowering diet score identified a plant-based dietary pattern, as proved by strong direct correlation with intake of fruits, vegetables, whole grains, and legumes, and by the inverse association with consumption of food from animal sources (i.e., meat and dairy products). Nonetheless, the cholesterol-lowering diet score showed a mild correlation with the hPDI. This is not totally unexpected since the two indexes focus on different dietary aspects deriving from a different rationale and a different construction. The hPDI scores the consumption of 18 foods, according to their adherence to a plant-based diet [28]. Conversely, the cholesterol-lowering diet considered seven specific foods and nutrients with known capability in modifying the serum lipid profile [11,12,14]. The two indexes have only one food in common (i.e., legumes), whereas they have a different approach to consider the food from vegetal sources (i.e., non-cellulosic polysaccharide soluble fibers and seed/corn oil in the cholesterol-lowering diet score vs. fruits, vegetables, vegetable oil, and fruit juices in the hPDI), food from animal sources (dietary MUFAs, SFAs, and cholesterol vs. animal fat, dairy, eggs, fish, and meat), and carbohydrate quality (i.e., glycemic index vs. whole grains, refined grains, potatoes, and sweets and desserts).

A number of potential limitations has to be acknowledged. Firstly, the score was created assigning 1 point when the dietary requirement was met, 0 otherwise. This may have introduced misclassification since it did not weight each dietary indicator according to its impact on serum cholesterol level. However, differently from clinical predictive and prognostic scores, the intent of our score was to evaluate associations with outcomes at a group level rather than at an individual level; therefore, potential classification bias is negligible. Notably, this approach is quite common and widely accepted in etiological and observational studies. Indeed, similar approaches were adopted to calculate MEDAS [26,27] and hPDI [28], and to create scores for adherence to the Mediterranean diet (MDS) [35], to healthy eating (HEI-2015 and HEI-2020) [36,37], to Dietary Approaches to Stop Hypertension (DASH) [38], and to the dietary recommendation of the World Cancer Research Fund/American Institute for Cancer Research [39]. Secondly, the DEDiCa trial included women who previously underwent surgery for breast cancer, and patients’ diet may have changed after cancer diagnosis or surgery. To overcome this potential bias, dietary habits were assessed at least three months after surgery and prior to any lifestyle modification foreseen by the DEDiCa study protocol. In addition, the present study considered women only, so gender-specific effects cannot be evaluated. Furthermore, although blood samples were collected according to the standardized study protocol, biochemical analyses were centralized in the coordinating center, and methods were standardized by the manufacturer, technical imprecisions were possible. However, the aim of the present analysis was a proof of principle, i.e., to verify the ability of the cholesterol-lowering diet score to capture specific serum lipid trends. Therefore, this aim can be pursued in any kind of population, as long as dietary information and serum lipid profile are available. Nonetheless, future investigations on a male population would add relevant insights. Conversely, the results of the present analysis were strengthened by the use of data from a clinical trial. Data were collected prospectively, and usual diet was assessed for seven consecutive days just prior to blood collection. Patients were asked to report daily food consumption in a detailed food diary, which was reviewed by study dietitians and centrally converted into nutrients by a team of trained nutritionists. Further, blood samples were collected according to standardized study protocols, and biochemical analyses were centralized in the coordinating center [17].

## 5. Conclusions

The present analyses showed that the cholesterol-lowering diet score is inversely associated with serum total cholesterol, LDL-C, and triglyceride levels. Although the average reduction in serum levels across the level of cholesterol-lowering diet score was small, it is worth noting that the proportion of women outside the recommend ranges of serum total cholesterol, LDL-C, and triglycerides greatly declined with increasing adherence to the cholesterol-lowering diet. This provides experimental evidence that this score is capable of detecting a dietary pattern that affects serum lipid profile, especially LDL-C.

## Figures and Tables

**Table 1:** Serum lipid profile (mg/dL) according to baseline characteristics of women with breast cancer enrolled in DEDiCa study.

Characteristics	Patients	(%)	Serum Lipid Profile (mg/dL)—Median (Q_1_–Q_3_)			
			Total Cholesterol	HDL-C	LDL-C	Triglycerides
Age						
<50 years	146	(50.0)	182 (164–207)	57 (47–70)	110 (92–131)	79 (57–105)
≥50 years	146	(50.0)	190 (171–216)	54 (46–63)	121 (105–145)	104 (79–134)
Kruskal–Wallis test			*p* = 0.04	*p* = 0.08	*p* < 0.01	*p* < 0.01
Tobacco smoking						
Never	131	(44.9)	181 (165–206)	56 (47–68)	111 (94–131)	82 (60–109)
Former/Current	161	(55.1)	189 (171–216)	54 (46–66)	121 (102–143)	96 (71–127)
Kruskal–Wallis test			*p* = 0.03	*p* = 0.33	*p* = 0.02	*p* < 0.01
Alcohol drinking						
Never	197	(67.5)	185 (167–208)	54 (46–65)	117 (96–136)	91 (68–122)
Former/Current	95	(32.5)	184 (170–220)	57 (46–69)	116 (98–142)	87 (63–121)
Kruskal–Wallis test			*p* = 0.40	*p* = 0.11	*p* = 0.54	*p* = 0.46
Body mass index						
<25 kg/m^2^	115	(39.4)	180 (161–200)	61 (50–73)	107 (92–129)	67 (53–87)
25 to <30 kg/m^2^	89	(30.5)	186 (167–211)	54 (46–66)	119 (94–137)	94 (79–124)
≥30 kg/m^2^	88	(30.1)	196 (174–220)	52 (43–57)	128 (111–154)	112 (95–146)
Kruskal–Wallis test for trend			*p* < 0.01	*p* < 0.01	*p* < 0.01	*p* < 0.01
Physical activity						
<5000 steps/day	139	(47.6)	189 (171–219)	53 (44–63)	120 (100–147)	107 (77–139)
≥5000 steps/day	153	(52.4)	181 (166–206)	56 (48–70)	114 (96–133)	80 (61–104)
Kruskal-Wallis test			*p* = 0.04	*p* = 0.01	*p* = 0.05	*p* < 0.01
Breast cancer stage						
I	81	(27.7)	185 (164–207)	56 (45–67)	118 (96–137)	86 (60–123)
IIA-IIB	165	(56.5)	184 (168–211)	53 (46–66)	116 (97–136)	90 (67–120)
IIIA-IIIC	46	(15.8)	191 (170–217)	56 (48–70)	120 (106–141)	90 (68–114)
Kruskal–Wallis test			*p* = 0.61	*p* = 0.49	*p* = 0.66	*p* = 0.98

HDL-C: high-density-lipoprotein cholesterol; LDL-C: low-density-lipoprotein cholesterol; Q_1_: first quartile; Q_3_: third quartile.

**Table 2:** Serum cholesterol levels (mg/dL) according to dietary indicators.

Dietary Indicators (Cut-Off)	Score Points	Patients	Serum Lipid Profile (mg/dL)—Median (Q_1_–Q_3_)			
			Total Cholesterol	HDL-C	LDL-C	Triglycerides
Non-cellulosic polysaccharides soluble fibers ^a^						
<3.6 g/day	0	215	185 (167–212)	54 (45–68)	118 (98–140)	89 (66–124)
≥3.6 g/day	1	77	179 (169–208)	57 (50–67)	108 (93–132)	92 (66–114)
Kruskal–Wallis test			*p* = 0.39	*p* = 0.19	*p* = 0.09	*p* = 0.63
Monounsaturated fatty acids						
<17.9% of kcal/day	0	219	186 (169–214)	54 (46–66)	117 (99–142)	93 (69–122)
≥17.9% of kcal/day	1	73	184 (164–199)	55 (46–70)	117 (90–129)	84 (61–121)
Kruskal–Wallis test			*p* = 0.11	*p* = 0.41	*p* = 0.08	*p* = 0.14
Legumes						
<22.9 g/day	0	218	186 (169–213)	54 (45–66)	119 (97–139)	92 (68–124)
≥22.9 g/day	1	74	181 (167–208)	56 (50–68)	110 (97–137)	83 (63–112)
Kruskal–Wallis test			*p* = 0.29	*p* = 0.07	*p* = 0.25	*p* = 0.16
Saturated fatty acids						
<8.3% of kcal/day	1	73	180 (162–203)	56 (47–65)	111 (93–133)	85 (65–113)
≥8.3% of kcal/day	0	219	186 (170–213)	54 (46–68)	119 (98–140)	89 (68–123)
Kruskal–Wallis test			*p* = 0.11	*p* = 0.89	*p* = 0.11	*p* = 0.41
Seeds or corn oil ^b^						
<2.8 g/day per 1000 kcal	0	219	185 (169–212)	55 (46–67)	116 (97–140)	91 (66–122)
≥2.8 g/day per 1000 kcal	1	73	185 (167–203)	56 (47–68)	118 (98–130)	88 (67–116)
Kruskal–Wallis test			*p* = 0.50	*p* = 0.51	*p* = 0.84	*p* = 0.51
Dietary cholesterol						
<200 mg/day	1	226	185 (168–208)	54 (46–66)	117 (96–136)	89 (65–122)
≥200 mg/day	0	66	189 (170–221)	57 (47–70)	118 (100–143)	92 (67–121)
Kruskal–Wallis test			*p* = 0.35	*p* = 0.27	*p* = 0.50	*p* = 0.97
Glycemic index (GI) ^c^						
<77.1	1	71	185 (168–206)	58 (49–69)	116 (97–135)	89 (66–107)
≥77.1	0	221	185 (168–212)	54 (46–66)	118 (97–140)	90 (66–126)
Kruskal–Wallis test			*p* = 0.70	*p* = 0.12	*p* = 0.68	*p* = 0.33
Cholesterol-lowering diet score						
0–1		80	193 (169–223)	54 (46–65)	122 (101–151)	91 (69–124)
2		97	186 (171–207)	54 (45–70)	117 (97–137)	86 (61–127)
3		68	181 (160–207)	54 (46–61)	117 (99–132)	104 (82–142)
4–6		47	177 (163–197)	61 (53–73)	107 (91–124)	70 (55–92)
Jonckheere–Terpstra test			*p* < 0.01	*p* = 0.13	*p* < 0.01	*p* = 0.09

HDL-C: high-density-lipoprotein cholesterol; LDL-C: low-density-lipoprotein cholesterol; Q_1_: first quartile; Q_3_: third quartile. ^a^ As a proxy of viscose fibers. ^b^ As a proxy of phytosterol. ^c^ White bread scale (multiply by 0.71 to convert GI values to the glucose scale).

**Table 3:** *β* coefficients and corresponding standard error (SE) from the quantile regression model predicting serum lipid profile.

Characteristics	Serum Lipid Profile—*β* (SE)			
	Total Cholesterol	HDL-C	LDL-C	Triglycerides
Age (reference: <50 years)				
≥50 years	7.0 (5.2)	−0.7 (2.8)	11.0 (4.9)	11.0 (5.2)
Student’s *t*-test	*p* = 0.18	*p* = 0.81	*p* = 0.03	*p* = 0.03
Tobacco smoking (reference: Never)				
Former/current	5.0 (4.7)	0.3 (2.3)	4.5 (5.2)	7.0 (5.4)
Student’s *t*-test	*p* = 0.29	*p* = 0.89	*p* = 0.39	*p* = 0.20
Body mass index (reference: <25 kg/m^2^)				
25 to <30 kg/m^2^	4.0 (5.8)	−5.7 (3.4)	3.5 (7.0)	21.0 (5.7)
≥30 kg/m^2^	13.0 (5.9)	−8.0 (3.5)	14.0 (5.9)	35.0 (6.0)
Student’s *t*-test	*p* = 0.06	*p* = 0.02	*p* = 0.01	*p* < 0.01
Physical activity (reference: <5000 steps/day)				
≥5000 steps/day	−3.0 (4.7)	3.0 (2.4)	−3.5 (4.2)	−5.0 (5.0)
Student’s *t*-test	*p* = 0.52	*p* = 0.21	*p* = 0.41	*p* = 0.32
Cholesterol-lowering diet score (reference: 0 to 1)				
2	−6.0 (6.5)	−0.7 (3.5)	−12.5 (6.0)	−3.0 (6.6)
3	−14.0 (7.4)	0.7 (3.2)	−13.5 (5.5)	−1.0 (6.0)
4–6	−12.0 (7.2)	4.0 (4.0)	−18.5 (6.3)	−14.0 (5.5)
Student’s *t*-test for trend	*p* = 0.02	*p* = 0.36	*p* < 0.01	*p* = 0.04

HDL-C: high-density-lipoprotein cholesterol; LDL-C: low-density-lipoprotein cholesterol.

**Table 4:** Median daily intake (g) of selected food groups, with corresponding first and third quartile (Q_1_–Q_3_), according to cholesterol-lowering diet score.

Food Group (g Day)	Cholesterol-Lowering Diet Score (Score Points)				
	0–1	2	3	4–6	Kruskal-Wallis Test
	Median (Q_1_–Q_3_)	Median (Q_1_–Q_3_)	Median (Q_1_–Q_3_)	Median (Q_1_–Q_3_)	
Fruits	126 (78–193)	139 (92–220)	159 (77–247)	244 (161–331)	*p* < 0.01
Vegetables	101 (64–148)	125 (74–193)	131 (90–174)	183 (123–283)	*p* < 0.01
Refined grains	115 (80–145)	102 (73–136)	88 (71–108)	90 (54–127)	*p* < 0.01
Whole grains	8 (0–31)	15 (0–30)	9 (0–33)	27 (4–60)	*p* < 0.01
Potatoes	14 (0–34)	14 (0–29)	11 (0–26)	9 (0–28)	*p =* 0.51
Legumes	9 (1–17)	13 (6–20)	18 (7–36)	32 (20–41)	*p* < 0.01
Meat	74 (45–104)	63 (39–82)	64 (40–88)	49 (28–70)	*p* < 0.01
Fish	30 (14–49)	23 (11–49)	30 (14–54)	40 (18–76)	*p* = 0.05
Dairy products	119 (51–186)	85 (27–189)	42 (15–131)	29 (12–87)	*p* < 0.01
Olive oil	14 (10–19)	16 (10–22)	19 (13–23)	20 (14–23)	*p* < 0.01

## Data Availability

The data presented in this study are available for research purposes on request from the corresponding author. The data are not publicly available since data analyses are still ongoing.

## Supplementary Materials

The following supporting information can be downloaded at: https://www.mdpi.com/article/10.3390/nu16040495/s1, Table S1: Sensitivity analyses of the association between cholesterol-lowering diet score and serum lipid profile.
