# Supplementary material for: A Plant-Based Cholesterol-Lowering Diet Score Correlates with Serum LDL-Cholesterol Levels
Source: Nutrients. 2024 Feb 8;16(4):495. doi: 10.3390/nu16040495 (PMC10892311; doi:10.3390/nu16040495)
Supplement: Supplementary file 1 [file nutrients-16-00495-s001.zip › nutrients-2806084-supplementary.pdf]

Table S1. Sensitivity analyses on serum cholesterol levels (mg/dL) according to cholesterol-lowering diet score excluding women with metabolic disorders.

Dietary indicators (Cut-off)	n	Serum lipid profile (mg/dL) - Median (Q_1-Q_3)			
		**Total cholesterol**	**HDL-C**	**LDL-C**	**Triglycerides**
Overall (n=292)					
Cholesterol-lowering diet score					
0-1	80	193 (169-223)	54 (46-65)	122 (101-151)	91 (69-124)
2	97	186 (171-207)	54 (45-70)	117 (97-137)	86 (61-127)
3	68	181 (160-207)	54 (46-61)	117 (99-132)	104 (82-142)
4-6	47	177 (163-197)	61 (53-73)	107 (91-124)	70 (55-92)
Jonkheere-Tepstra test		$p < 0.01$	$p = 0.13$	$p < 0.01$	$p = 0.09$
Excluding women with diabetes mellitus (n=284)					
Cholesterol-lowering diet score					
0-1	79	190 (168-222)	54 (46-65)	121 (101-148)	91 (69-122)
2	95	186 (171-207)	54 (45-70)	117 (98-137)	86 (61-127)
3	65	181 (159-207)	53 (46-61)	116 (99-132)	103 (82-142)
4-6	45	177 (167-198)	61 (53-73)	107 (91-125)	70 (54-91)
Jonkheere-Tepstra test		$p = 0.01$	$p = 0.11$	$p = 0.01$	$p = 0.09$
Excluding women with hyperglycemia (n=281)					
Cholesterol-lowering diet score					
0-1	78	190 (166-221)	54 (45-65)	121 (100-147)	89 (68-123)
2	93	186 (171-207)	54 (46-70)	116 (97-132)	86 (60-126)
3	65	181 (159-207)	53 (46-61)	114 (98-132)	102 (82-142)
4-6	45	177 (167-198)	61 (53-73)	107 (91-125)	70 (54-91)
Jonkheere-Tepstra test		$p = 0.01$	$p = 0.10$	$p = 0.01$	$p = 0.09$
Excluding women with diabetes mellitus or hyperglycemia (n=280)					
Cholesterol-lowering diet score					
0-1	78	190 (166-221)	54 (45-65)	121 (100-147)	89 (68-123)
2	93	186 (171-207)	54 (46-70)	116 (97-137)	86 (60-126)
3	64	181 (159-207)	53 (46-61)	115 (99-132)	103 (82-142)
4-6	45	177 (167-198)	61 (53-73)	107 (91-125)	70 (54-91)
Jonkheere-Tepstra test		$p = 0.02$	$p = 0.10$	$p = 0.02$	$p = 0.10$
